# First Evidence of Bovine Leukemia Virus in *Culicoides* Biting Midges and the Stable Fly *Stomoxys calcitrans* Collected on Cattle Farms in Poland

**DOI:** 10.1155/tbed/7012598

**Published:** 2026-06-25

**Authors:** Aneta Pluta, Joanna Kowalik, Wojciech Rożek, Małgorzata Kwaśnik, Anna Ziętek-Barszcz, Jacek Kuźmak

**Affiliations:** ^1^ Department of Virology and Viral Animal Diseases, National Veterinary Research Institute, Puławy, Poland, piwet.pulawy.pl; ^2^ Department of Research Support, National Veterinary Research Institute, Puławy, Poland, piwet.pulawy.pl

**Keywords:** BLV detection in insects, bovine leukemia virus (BLV), *Culicoides* spp., hematophagous insects, mechanical transfer of BLV, phylogenetic analysis, *Stomoxys calcitrans*

## Abstract

Bovine leukemia virus (BLV) is a cell‐associated retrovirus of cattle transmitted mainly through the transfer of infected lymphocytes, but the possible role of hematophagous insects in its epidemiology remains incompletely understood. This study investigated BLV DNA in field‐collected *Culicoides* biting midges and *Stomoxys calcitrans* from cattle farms in Poland. Insects were collected on four farms in northeastern Poland. *Culicoides* spp. were identified morphologically, classified by gonotrophic status, and pooled by species and status, whereas *S. calcitrans* were analyzed individually. DNA quality was verified by PCR targeting insect markers. Bovine host DNA was detected by PCR targeting mitochondrial cytochrome b in *Culicoides* and prepronociceptin (PNOC) in *S. calcitrans*. BLV proviral DNA was detected by TaqMan real‐time PCR targeting the *pol* gene, followed by nested PCR and sequencing of a 400 bp fragment of the *env* gene. BLV DNA was detected in 20 of 138 *Culicoides* pools (14.5%). BLV copy numbers ranged from 2 to 19,000 copies per 500 ng of total DNA. All BLV‐positive *Culicoides* pools were also positive for bovine cytochrome b DNA. Among the *pol*‐positive pools, 18 were positive in nested *env* PCR, and 15 yielded high‐quality sequences. One *S. calcitrans* specimen was also positive in both BLV assays. Sequences obtained from insects were identical to those from infected cattle, and phylogenetic analysis assigned them to genotypes G4 and G7, the predominant BLV genotypes circulating in Poland. These findings provide field‐based molecular evidence that *Culicoides* biting midges and *S. calcitrans* can carry bovine blood and BLV DNA under natural farm conditions, supporting their possible role in the mechanical transfer of BLV between cattle.

## 1. Introduction

Bovine leukemia virus (BLV), an oncogenic deltaretrovirus and the causative agent of enzootic bovine leukosis (EBL), remains one of the most widespread viral infections of cattle worldwide and continues to cause economic losses associated with reduced productivity, trade restrictions, and premature culling. Most infected animals remain asymptomatic, but a proportion develop persistent lymphocytosis, and a smaller fraction progress to the malignant form of EBL [1]. BLV causes a lifelong infection in which viral DNA persists as a provirus integrated into the genome of host cells [2]. Because the virus is highly cell‐associated, transmission depends primarily on the transfer of infected cells, especially lymphocytes, rather than on cell‐free virus. Consequently, even small amounts of blood containing infected lymphocytes may be epidemiologically relevant [3]. Under field conditions, BLV spreads mainly through horizontal transmission involving management‐related blood transfer, including reuse of needles, rectal palpation sleeves, dehorning, or other procedures that permit transfer of infected lymphocytes between animals. However, the possible role of hematophagous insects in BLV epidemiology has attracted attention for decades, because interrupted feeding could mechanically transfer infected blood from one host to another. Experimental and field studies have implicated blood‐sucking flies, particularly tabanids and stable flies, as potential mechanical vectors, although their contribution under natural farm conditions remains difficult to quantify [4]. Among biting Diptera, *Culicoides* biting midges are of particular veterinary importance because they are abundant around livestock, frequently attack cattle, and are proven vectors of several major ruminant viruses, including bluetongue and Schmallenberg virus [5, 6]. In Europe, renewed attention to *Culicoides* followed the emergence and spread of these pathogens, and numerous studies have shown that common Palearctic species readily feed on domestic ruminants, especially cattle. Importantly, detection of viral nucleic acids in field‐collected *Culicoides* does not by itself prove biological vector competence, because viral material may originate from an ingested blood meal. Nevertheless, such findings are epidemiologically informative, particularly for pathogens that may be transferred mechanically during interrupted feeding [7]. The evidence linking BLV to *Culicoides* remains extremely limited. To date, only isolated reports have described BLV detection in *Culicoides* pools, including a study from Türkiye reporting genotype 1 BLV in a *C. schultzei* pool. By contrast, molecular and phylogenetic studies in cattle from Poland and neighboring countries have shown that BLV strains circulating in this region belong predominantly to genotypes G4 and G7 [8, 9], providing a useful epidemiological background for comparison of insect‐ and cattle‐derived viral sequences [10]. Because *Culicoides* midges are abundant in cattle environments, frequently feed on bovine hosts, and may carry viral nucleic acids acquired during blood feeding, they represent a plausible but poorly investigated component of the BLV transmission ecology. In addition, the stable fly, *Stomoxys calcitrans*, is a well‐recognized blood‐feeding pest of cattle and has long been considered a candidate mechanical vector of BLV. Clarifying whether BLV DNA can be detected in these insects under natural farm conditions may help to better understand possible noniatrogenic routes of virus circulation within infected herds [4].

In 2017, following more than 30 years of the EBL eradication program, Poland was officially declared EBL‐free by Commission Implementing Decision (EU) 2017/888 of May 22, 2017 (https://eur-lex.europa.eu/eli/dec_impl/2017/888/oj/eng). Consequently, all Polish regions (voivodeships) implemented a surveillance program based on seroepidemiological monitoring within respective 5‐year regional plans. Nevertheless, the presence of persistent infection clusters has been confirmed in the Warmian–Masurian Voivodeship in northeastern Poland, based on regular serological testing by ELISA. Between 2020 and 2025, a total of 44 new outbreaks were recorded in this region out of 70 reported nationwide, accounting for ~63% of all cases. Similarly, the proportion of infected animals and herds in this period reached 54.6% and 61.9%, respectively. These data clearly indicate that, despite the implementation of numerous control measures provided by Commission Delegated Regulation (EU) 2020/689 (https://eur-lex.europa.eu/eli/reg_del/2020/689/oj/eng), a persistent BLV infection cluster remains in this region. One possible explanation for this situation may be the uncontrolled transmission of BLV by blood‐sucking insects. This hypothesis is supported by the specific environmental conditions of the Warmia and Masuria region, including a high density of lakes and forests, extensive pasture systems, a long grazing season, and a well‐developed dairy cattle industry. Therefore, the aim of the present study was to investigate the presence of BLV DNA in *Culicoides* biting midges and *S. calcitrans* collected from cattle farms in Poland, with particular emphasis on their potential role in BLV transmission.

## 2. Materials and Methods

### 2.1. Sample Collection

**Figure 1:**
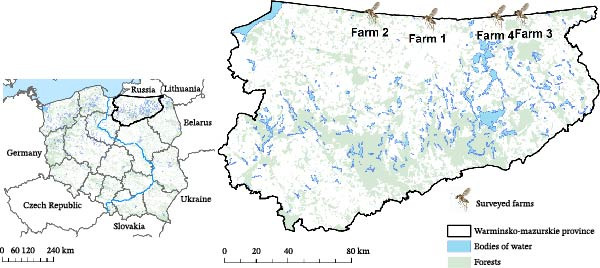
Geographic location of the cattle farm sampling sites in the Warmian–Masurian Province, northeastern Poland.

Insect samples were collected on four cattle farms located in the Warmian–Masurian Province in northeastern Poland. The location of the sampling sites is shown in Figure 1, with coordinates as follows: Farm 1 (54.3224°N, 21.1564°E), Farm 2 (54.3676°N, 20.5997°E), Farm 3 (54.3323°N, 22.0470°E), and Farm 4 (54.3262°N, 22.0465°E). The herds differed in size, management system, and history of infection with BLV (Table 1). The surrounding landscape was characterized by numerous wetlands, pastures, and water bodies, providing favorable habitats for hematophagous insects. Sampling was conducted during the insect activity season between June and July 2023. The insects were collected using two UV light traps of the Onderstepoort type (Onderstepoort Veterinary Institute, South Africa) placed inside livestock buildings and in their immediate surroundings near cattle housing to capture biting midges of the genus *Culicoides*. In addition, three blue sticky boards (30 cm × 60 cm; P.P.H. Medchem, Poland) were installed at the same locations to capture stable flies *S. calcitrans*. The traps were suspended at a height of ~1.5–2 m above ground level and were operated overnight for ~24 h. All insects captured during the 24‐h trapping period at each farm were included in the study.

**Table 1:** Characteristics of the four cattle farms, herd management, and environmental conditions during hematophagous insect collection.

Parameter	Farm 1	Farm 2	Farm 3	Farm 4
Location (voivodeship, county)	Warmian–Masurian, Bartoszyce county	Warmian–Masurian, Bartoszyce county	Warmian–Masurian, Gołdap county	Warmian–Masurian, Gołdap county
Date of insect collection	6–7 June 2023	24–25 July 2023	4–5 July 2023	4–5 July 2023
Surrounding landscape	Wetlands, pastures and water bodies in agricultural landscape	Wetlands, drainage ditches and pastures; large bird colonies	Agricultural landscape with wetlands and pastures	Agricultural landscape with wetlands and pastures
Herd size (number of cattle)	343	221	12	56
Cattle type	Dairy herd	Beef herd	Beef‐type crossbred herd	Mixed herd
Breed composition	Holstein–Friesian crossbred	Limousin	Limousin crossbred	Holstein–Friesian, Limousin crossbred, Simmental, Montbéliarde
Housing system	Tie‐stall	Free‐stall	Tie‐stall	Tie‐stall
Grazing during insect season	Yes—dry cows and heifers; No—lactating cows	Yes	Yes	Yes
Year of first BLV detection	2010	2019	2023	2023
BLV‐seropositive animals in May–June 2023	Yes—16 cows (4.7% seropositive)	No (one suspected case later tested negative; no seroconversion)	Yes—7 cows (58.3% seropositive)	Yes—10 cows (17.9% seropositive)
Introduction of new animals (last 5 years)	No (last in 2012)	Yes—28 animals from 12 herds	No	No
Number and type of insect traps used	2 Onderstepoort traps + 3 sticky traps			
Total *Culicoides* collected	1387	520	97	414
Number of *Culicoides* pools	75	33	6	24
Number of *Stomoxys calcitrans* collected	9	28	39	70

In addition to insect sampling, the infection status of cattle was assessed on all farms. All these farms were included in the regional surveillance programme for EBL in accordance with the Commission Delegated Regulation (EU) 2020/689. Serum samples were tested for antibodies against BLV using the IDEXX Leukosis Blocking ELISA test performed by the Veterinary Hygiene Laboratory in Olsztyn. Seropositive cattle were detected on farm numbers 1, 3, and 4, while Farm 2 was officially declared EBL‐free since 2021 (Table 1). Blood samples from ELISA‐positive animals were sent to the National Veterinary Research Institute (NVRI) in Puławy for confirmatory testing. All positive samples and corresponding DNA extracts were archived at the institute, from which 11 DNA samples were selected for further study.

### 2.2. Insect Identification and Pooling

Immediately after collection, the insects were transferred into 70% ethanol and transported to the laboratory, where they were stored at 4°C until processing. Specimens were subsequently sorted and identified to the species level under a stereoscopic microscope (Olympus) using available taxonomic keys (Table 2). Specimens initially identified morphologically as *C. obsoletus* were treated as members of the *obsoletus* complex, as females of closely related species within this complex cannot be reliably distinguished by morphology alone. *S. calcitrans* specimens collected on blue sticky boards were identified morphologically using a stereoscopic microscope, based on characteristic external features and published identification keys [11], and were analyzed individually. The number of *S. calcitrans* specimens collected on each farm is provided in Table 1.

**Table 2:** Species composition and abundance of *Culicoides* biting midges collected at four cattle farms in Poland.

Species	Farm 1 (*n* = 1387)	Farm 2 (*n* = 520)	Farm 3 (*n* = 97)	Farm 4 (*n* = 414)	Total (*n* = 2418)
*C. punctatus*	687 (49.5%)	139 (26.7%)	6 (6.2%)	113 (27.3%)	945 (39.1%)
*C. obsoletus/C. scoticus*	337 (24.3%)	295 (56.7%)	56 (57.7%)	176 (42.5%)	864 (35.7%)
*C. achrayi*	299 (21.6%)	2 (0.4%)	1 (1.0%)	3 (0.7%)	305 (12.6%)
*C. pallidicornis*	36 (2.6%)	38 (7.3%)	29 (29.9%)	70 (16.9%)	173 (7.2%)
*C. fascipennis*	28 (2.0%)	5 (1.0%)	—	—	33 (1.4%)
*C. pulicaris*	—	22 (4.2%)	4 (4.1%)	14 (3.4%)	40 (1.7%)
*C. circumscriptus*	—	11 (2.1%)	1 (1.0%)	18 (4.3%)	30 (1.2%)
*C. newsteadi*	—	—	—	13 (3.1%)	13 (0.5%)
*C. grisescens*	—	8 (1.5%)	—	1 (0.2%)	9 (0.4%)
*C. furcillatus*	—	—	—	3 (0.7%)	3 (0.1%)
*C. riethi*	—	—	—	3 (0.7%)	3 (0.1%)

*Note:* Percentages for each farm represent the proportion of a given species relative to the total number of *Culicoides* specimens collected at that farm.

Along with taxonomic identification, *Culicoides* specimens were sorted according to gonotrophic forms: nulliparous, parous, blood‐fed, gravid females, and males (Table 3). Nulliparous females were identified by a pale, transparent abdomen, whereas parous females had a pink abdomen, indicating at least one completed gonotrophic cycle. Blood‐fed females were recognized by the presence of a visible blood meal, and gravid females by mature eggs visible through the abdominal wall. Females with visible mature eggs were classified as gravid, regardless of previous parity status.

**Table 3:** Species composition and gonotrophic status of *Culicoides* biting midges collected at four cattle farms.

Farm	Species	Nulliparous	Parous	Blood‐fed	Gravid	Male	Total
1	*C. punctatus*	236 (17.0%)	240 (17.3%)	164 (11.8%)	—	47 (3.4%)	687 (49.5%)
	*C. achrayi*	161 (11.6%)	26 (1.9%)	110 (7.9%)	2 (0.1%)	—	299 (21.6%)
	*C. obsoletus/C. scoticus*	80 (5.8%)	98 (7.1%)	80 (5.8%)	79 (5.7%)	—	337 (24.3%)
	*C. pallidicornis*	25 (1.8%)	5 (0.4%)	6 (0.4%)	—	—	36 (2.6%)
	*C. fascipennis*	20 (1.4%)	3 (0.2%)	5 (0.4%)	—	—	28 (2.0%)
2	*C. obsoletus/C. scoticus*	100 (19.2%)	120 (23.1%)	45 (8.7%)	29 (5.6%)	1 (0.2%)	295 (56.7%)
	*C. punctatus*	60 (11.5%)	60 (11.5%)	16 (3.1%)	1 (0.2%)	2 (0.4%)	139 (26.7%)
	*C. pallidicornis*	14 (2.7%)	13 (2.5%)	1 (0.2%)	10 (1.9%)	—	38 (7.3%)
	*C. pulicaris*	8 (1.5%)	8 (1.5%)	5 (1.0%)	—	1 (0.2%)	22 (4.2%)
	*C. fascipennis*	5 (1.0%)	—	—	—	—	5 (1.0%)
	*C. circumscriptus*	—	5 (1.0%)	—	6 (1.2%)	—	11 (2.1%)
	*C. grisescens*	6 (1.2%)	2 (0.4%)	—	—	—	8 (1.5%)
	*C. achrayi*	—	1 (0.2%)	—	1 (0.2%)	—	2 (0.4%)
3	*C. obsoletus/C. scoticus*	—	40 (41.2%)	6 (6.2%)	8 (8.2%)	2 (2.1%)	56 (57.7%)
	*C. pallidicornis*	15 (15.5%)	14 (14.4%)	—	—	—	29 (29.9%)
	*C. punctatus*	2 (2.1%)	1 (1.0%)	3 (3.1%)	—	—	6 (6.2%)
	*C. pulicaris*	2 (2.1%)	2 (2.1%)	—	—	—	4 (4.1%)
	*C. achrayi*	—	—	—	—	1 (1.0%)	1 (1.0%)
	*C. circumscriptus*	—	—	—	1 (1.0%)	—	1 (1.0%)
4	*C. obsoletus/C. scoticus*	36 (8.7%)	60 (14.5%)	3 (0.7%)	60 (14.5%)	17 (4.1%)	176 (42.5%)
	*C. punctatus*	52 (12.6%)	40 (9.7%)	12 (2.9%)	—	9 (2.2%)	113 (27.3%)
	*C. pallidicornis*	40 (9.7%)	30 (7.2%)	—	—	—	70 (16.9%)
	*C. circumscriptus*	1 (0.2%)	—	—	17 (4.1%)	—	18 (4.3%)
	*C. pulicaris*	—	14 (3.4%)	—	—	—	14 (3.4%)
	*C. newsteadi*	6 (1.4%)	7 (1.7%)	—	—	—	13 (3.1%)
	*C. achrayi*	—	3 (0.7%)	—	—	—	3 (0.7%)
	*C. furcillatus*	—	3 (0.7%)	—	—	—	3 (0.7%)
	*C. riethi*	—	3 (0.7%)	—	—	—	3 (0.7%)
	*C. grisescens*	1 (0.2%)	—	—	—	—	1 (0.2%)

*Note:* Percentages are calculated relative to the total number of *Culicoides* collected at each farm.

Individuals were pooled in groups of 5–20 specimens per pool to obtain a sufficient DNA concentration for PCR‐based analyses. Pools of *Culicoides* specimens were prepared separately for each farm according to the species and gonotrophic status (Table 4). Specimens represented by small numbers of individuals (*n* ≤ 5) were not pooled and were excluded from the analysis.

**Table 4:** Number of *Culicoides* pools by species and gonotrophic forms at each farm.

Farm	Species	Nulliparous	Parous	Blood‐fed	Gravid	Male	Total pools
1	*C. punctatus*	21	12	9	0	3	**45**
	*C. achrayi*	8	2	6	0	0	**16**
	*C. obsoletus/C. scoticus*	4	5	4	4	0	**17**
	*C. pallidicornis*	2	1	1	0	0	**4**
	*C. fascipennis*	1	0	1	0	0	**2**
2	*C. obsoletus/C. scoticus*	5	6	3	2	0	**16**
	*C. punctatus*	3	3	1	0	0	**7**
	*C. pallidicornis*	1	1	0	1	0	**3**
	*C. pulicaris*	1	1	1	0	0	**3**
	*C. circumscriptus*	0	1	0	1	0	**2**
	*C. grisescens*	1	0	0	0	0	**1**
	*C. fascipennis*	1	0	0	0	0	**1**
3	*C. obsoletus/C. scoticus*	0	2	1	1	0	**4**
	*C. pallidicornis*	1	1	0	0	0	**2**
4	*C. obsoletus/C. scoticus*	2	3	0	3	1	**9**
	*C. punctatus*	3	2	1	0	1	**7**
	*C. pallidicornis*	2	2	0	0	0	**4**
	*C. pulicaris*	0	1	0	0	0	**1**
	*C. circumscriptus*	0	0	0	1	0	**1**
	*C. newsteadi*	1	1	0	0	0	**2**

*Note:* Bold indicates the overall summary result.

In total, 138 *Culicoides* pools were prepared for molecular analyses across the four farms: Farm 1: 75 pools; Farm 2: 33 pools; Farm 3: 6 pool; and Farm 4: 24 pools (Table 4). All pooled specimens were stored at −80°C until molecular analyses. Specimens of the stable fly *S. calcitrans* were collected in sterile microcentrifuge tubes individually and stored at −80°C until further study.

### 2.3. DNA Extraction

Total DNA was extracted from pools of *Culicoides* biting midges and from individual specimens of the *S. calcitrans* using the DNeasy Blood & Tissue Kit (Qiagen, Germany) according to the manufacturer’s protocol for purification of DNA from insects. Briefly, insect pools were homogenized in Lysing Matrix D tubes (MP Biomedicals, France) with 200 μL phosphate‐buffered saline (PBS) using a FastPrep‐24 5G bead mill homogenizer (MP Biomedicals, USA). Samples were then incubated with proteinase K and Buffer AL at 56°C to ensure complete lysis. After the addition of ethanol, the lysates were transferred to DNeasy Mini spin columns for purification. The columns were subsequently washed with Buffer AW1 and Buffer AW2, and DNA was eluted in 90 μL of Buffer AE. DNA concentration and purity were assessed spectrophotometrically using a NanoPhotometer (Implen, Germany). DNA concentration and purity values are provided in Supporting Information 1: Table S4. Extracted DNA samples were stored at −20°C until further molecular analyses.

### 2.4. PCR‐Based Verification of DNA Quality in Insect Samples

Conventional PCR assays were performed to verify the quality of DNA extracted from insect samples before subsequent molecular detection of BLV proviral DNA.

For *Culicoides* DNA samples, a fragment of the mitochondrial cytochrome oxidase I (COI) gene was amplified using primers C1‐J‐1718 and C1‐N‐2191 described by Simon et al. [12]. PCR amplification was performed using OptiTaq DNA polymerase (EURx, Poland) under the following cycling conditions: initial denaturation at 94°C for 3 min, followed by 37 cycles of 94°C for 1 min, 51°C for 1 min, and 72°C for 1 min, with a final extension at 72°C for 10 min. Primers used in this study are summarized in Supporting Information 2: Table S1. Amplification of the COI marker was not successful for all *Culicoides* species present in the analyzed material, likely due to the high sequence variability of the mitochondrial COI gene. An additional PCR assay targeting the 18S rRNA region was therefore performed using modified primers derived from NF1 and 18Sr2b [13], shortened in this study to improve the amplification efficiency. PCR amplification was performed using OptiTaq DNA polymerase (EURx, Poland) under the following cycling conditions: initial denaturation at 94°C for 3 min, followed by 35 cycles of 94°C for 30 s, 54°C for 45 s, and 68°C for 30 s, with a final extension at 72°C for 10 min.

For *S. calcitrans*, DNA quality was verified using the mitochondrial primer pair N1‐J‐12585 and LR‐N‐12866 described by Simon et al. [12], which amplifies a fragment of the mitochondrial 16S rRNA region. PCR amplification was performed using Taq DNA polymerase (EURx, Poland) under the following program: initial denaturation at 94°C for 3 min, followed by 33 cycles of 94°C for 1 min, 55°C for 1 min, and 72°C for 1 min, with a final extension at 72°C for 10 min. All PCR products were separated on 2% agarose gels stained with Simply Safe (EURx, Poland) and visualized under UV illumination. The presence of a clear single PCR amplicon of the expected size confirmed the integrity of insect DNA in the analyzed samples.

### 2.5. Detection of Bovine Host DNA in Insect Samples

Bovine host DNA in insect samples was detected by PCR targeting vertebrate markers used in blood meal analysis of hematophagous arthropods. In *Culicoides* biting midges, bovine DNA was detected by PCR amplification of the mitochondrial cytochrome b (*cyt b*) gene. Species‐specific primers (cytBovine_for and cytBovine_rev) were designed in the present study (Supporting Information 2: Table S1). Primer specificity was evaluated in silico using BLAST searches, and whole genome screening was performed with Geneious Prime 2025.1.2 (Biomatters Ltd., New Zealand). PCR amplification was performed using 200 ng of DNA and OptiTaq DNA polymerase (EURx, Poland) under the following cycling conditions: initial denaturation at 94°C for 3 min and by 33 cycles of 94°C for 1 min, 55°C for 1 min, and 72°C for 1 min, with a final extension at 72°C for 10 min.

Genome screening of the *S. calcitrans* mitochondrial genome revealed sequences similar to *cyt b*; therefore, this marker was not used for detection of bovine DNA in this species to avoid potential nonspecific amplification. Instead, bovine host DNA in *S. calcitrans* was detected using PCR targeting the nuclear prepronociceptin (*PNOC*) gene previously used in blood meal studies [14]. Primers PNOC_for and PNOC_rev were designed in the present study (Supporting Information 2: Table S1). PCR amplification was carried out under the same thermal cycling conditions as described for the *cyt b* assay. The presence of a clear single band on the electrophoregram was interpreted as confirmation of bovine host DNA in the analyzed insect samples.

### 2.6. Detection of BLV in Insect Samples by qPCR

Detection of BLV proviral DNA in insect samples was performed using a TaqMan real‐time PCR assay targeting the *pol* gene as previously described [8]. The assay employed the primer pair MRBLVL Fw and MRBLVR Rv together with the MRBLV probe (Supporting Information 2: Table S1). For quantification, the pBLV1 plasmid (NVRI, Puławy, Poland) containing a fragment of the BLV *pol* gene was used as a standard. Ten‐fold serial dilutions ranging from 1 × 10^6^ to 1 × 10^2^ copies μL^−1^ were prepared to generate the standard curve. The number of BLV proviral copies was calculated and expressed per 500 ng of total DNA.

### 2.7. PCR Amplification and Sequencing of 400 bp *env* Gene Fragment

A 444 bp fragment of the BLV *env* gene was amplified by nested PCR as previously described by Beier et al. [15]. The primer pairs *env*5032/*env*5608 and *env*5099/*env*5521 were used for the first and second rounds of amplification, respectively. Reactions were performed with 300 ng of genomic bovine or insect DNA using Q5 High‐Fidelity DNA Polymerase (New England Biolabs, USA). Thermal cycling consisted of an initial denaturation at 98°C for 30 s, followed by 40 cycles of 98°C for 10 s, primer annealing at 62°C (external primers) or 70°C (internal primers) for 30 s, and extension at 72°C for 40 s, with a final extension at 72°C for 5 min. PCR products were separated on a 1.5% agarose gel containing SimplySafe (EURx) and purified using a NucleoSpin Extract II Kit (Macherey‐Nagel GmbH & Co. KG). Purified amplicons were sequenced by Genomed SA (Warsaw, Poland) using the BigDye Terminator v3.1 kit and analyzed on a 3730xl DNA Analyzer (Thermo Fisher Scientific). The primers used for sequencing are listed in Supporting Information 2: Table S1.

### 2.8. Sequence and Phylogenetic Analysis

Raw sequencing reads were assembled in Geneious Prime v2025.1.2 (Biomatters Ltd., New Zealand) to generate consensus sequences. The resulting BLV sequences were deposited in the GenBank database under Accession Numbers PZ226357–PZ226361, PZ226363– PZ226366, PZ226368–PZ226369, and PZ226371–PZ226375 for sequences obtained from insect DNA and PZ226336–PZ226346 for sequences obtained from bovine DNA (Supporting Information 3: Table S2). Multiple sequence alignments were performed using the ClustalW algorithm implemented in Geneious Prime. All sequences were trimmed to a uniform length corresponding to the 400 bp fragment of the BLV *env* gene prior to phylogenetic analysis. In addition to the sequences generated in this study, representative BLV sequences corresponding to the 12 currently recognized genotypes were retrieved from GenBank and included in the analysis (Supporting Information 3: Table S2). Phylogenetic trees were constructed using the neighbor‐joining (NJ) method [16] with the Tamura–Nei model of nucleotide substitution [17]. The robustness of the inferred tree topology was evaluated by bootstrap analysis with 1000 replicates.

### 2.9. Statistical Analysis

The prevalence of BLV DNA in pooled *Culicoides* samples was estimated using the minimum detection rate (MDR) and the maximum likelihood estimate (MLE). MDR assumes that each positive pool contains at least one infected individual and was calculated as: MDR = (number of positive pools/total number of tested insects) × 1000, where the number of tested insects represents the total number of individuals included in all analyzed pools. Because MDR may underestimate infection prevalence in pooled samples, the MLE was additionally calculated. The likelihood function for pooled data was defined as *L*(*p*) = ∏(1 − *p*)*^n^ᵢ* × ∏[1 − (1 − *p*)*^n^ᵢ*], where *p* is the probability of infection and *nᵢ* is the number of insects in the *i*‐th pool. The infection rate was expressed per 1000 insects: MLE (per 1000 insects) = *p* × 1000. MLE values and 95% confidence intervals (95% CI) were estimated using PooledInfRate software (Version 4.0; Biggerstaff, CDC, USA). Both MDR and MLE were calculated for all *Culicoides* pools and for blood‐fed females only.

## 3. Results

### 3.1. DNA Quality Assessment and Detection of Bovine Blood in *Culicoides* Pools

Several *Culicoides* pools failed to yield detectable insect COI and 18S rRNA PCR amplicons and were therefore excluded from further study. Specifically, four DNA pools from Farm 1, three from Farm 2, none from Farm 3, and five from Farm 4 were removed from subsequent analyses (Supporting Information 4: Table S3). Detection of bovine DNA using the mitochondrial cytochrome *b* (*cyt b*) marker varied among farms. At Farm 1, bovine DNA was detected in 39 out of 71 pools (54.9%), representing the highest positivity among all sites. Positive pools were observed across multiple species, including *C. punctatus*, *C. achrayi*, and *C. obsoletus/C. scoticus*, as well as sporadically in *C. pallidicornis* and *C. fascipennis*. At Farm 2, bovine DNA was detected in 9 out of 30 pools (30.0%). Positive pools were mainly associated with *C. obsoletus/C. scoticus* and occurred less frequently in *C. punctatus*, *C. pulicaris*, *C. grisescens*, and *C. circumscriptus*. At Farm 3, bovine DNA was detected in 3 out of 6 pools (50%), indicating the lowest detection rate among all farms. At Farm 4, bovine DNA was detected in 5 out of 19 pools (26.3%), primarily in *C. obsoletus/C. scoticus*, with single positive pools also observed in *C. punctatus* and *C. pallidicornis*. Bovine DNA was detected in 39/71 pools from Farm 1, 9/30 pools from Farm 2, 3/6 pools from Farm 3, and 5/19 pools from Farm 4.

### 3.2. Detection and Molecular Characterization of BLV in *Culicoides* Pools

Real‐time PCR targeting the BLV *pol* gene yielded a total of 20 positive pools across all farms. The highest number of positive pools was observed at Farm 1 (*n* = 17), followed by Farm 4 (*n* = 2) and Farm 3 (*n* = 1), whereas no positive pools were detected at Farm 2 (*n* = 0) (Table 5).

**Table 5:** Detection of BLV DNA in *Culicoides* pools by qPCR and nested PCR.

Farm	Number of pools tested	qPCR‐positive pools (*n*, %)	Median BLV copies (range)	Nested PCR (*env*)‐positive pools (*n*, %)
1	71	17 (23.9)	100 (2–19,000)	15 (21.1)
2	30	0 (0)	—	0 (0)
3	6	1 (16.7)	220	1 (16.7)
4	19	2 (10.5)	22.5 (20–225)	2 (10.5)
**Total**	**126**	**20 (15.9)**	**48 (<1–19,000)**	**18 (14.3)**

*Note:* Bold indicates the final summary result.

**Figure 2:**
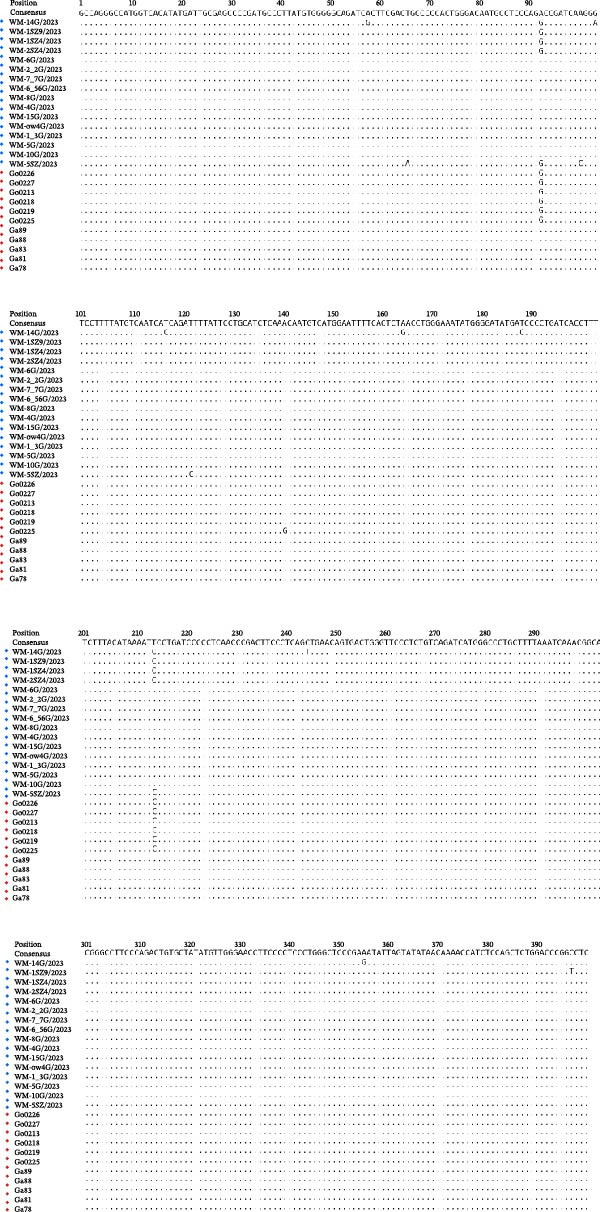
Alignment of BLV *env* sequences obtained from insects and infected cattle. Blue dots indicate sequences obtained from insects (WM isolates), whereas red dots indicate sequences obtained from cattle.

**Figure 3:**
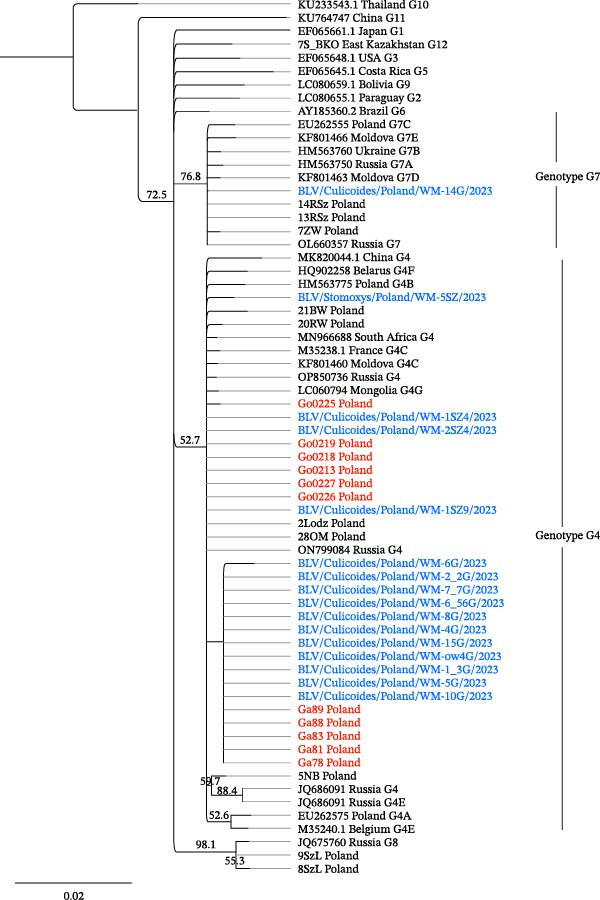
Neighbor‐Joining phylogenetic tree of BLV *env* sequences representing all 12 known genotypes (G1–G12). Sequences obtained from cattle in the Warmian–Masurian region (Poland) are highlighted in red, while sequences derived from *Culicoides* spp./*Stomoxys calcitrans* collected in the same region are shown in blue.

The number of BLV DNA copies ranged from 2 to 19,000 copies per 500 ng of total DNA. Importantly, all BLV‐positive pools were also positive for the bovine *cyt b* marker, confirming the presence of bovine DNA in all samples in which viral DNA was detected. Among the 20 pools positive in real‐time PCR targeting the BLV *pol* gene, 18 yielded a positive result in nested PCR targeting the *env* gene, as confirmed by the presence of a clear band of the expected size on agarose gel electrophoresis. Of these, 15 samples were successfully sequenced, and high‐quality nucleotide sequences were obtained, including 12 from Farm 1, 1 from Farm 3, and 2 from Farm 4 (Supporting Information 3: Table S2). The remaining samples either failed to produce sequences of sufficient quality and were therefore excluded from further analysis. The obtained sequences were aligned and are presented in Figure 2. In addition, all DNA samples (*n* = 11) derived from BLV‐infected cattle were successfully amplified and sequenced, and these sequences were also included in the alignment (Figure 2 and Supporting Information 3: Table S2). Comparative analysis of the 400‐bp BLV *env* fragment from Farm 1 showed that all cattle‐derived sequences were identical, and 11 of 12 insect‐derived sequences shared the same nucleotide pattern. In contrast, one insect‐derived sequence, BLV/Culicoides/Poland/WM‐14G/2023, differed by nine nucleotide substitutions and clustered within genotype G7, whereas all cattle‐derived sequences belonged to genotype G4; notably, this G7 sequence was identical to cattle‐derived sequences 14RSz and 13RSz originating from Warmian–Masurian, Szczytno County, Poland, collected in 2019 (Figure 3). This result was further confirmed by the cloning and sequencing of three independent clones, all of which were assigned to genotype G7. Although the cattle‐derived sequences obtained from Farm 1 in the present study belonged to genotype G4, only a subset of BLV‐positive cattle from this herd was sequenced. Therefore, the circulation of genotype G7 among other BLV‐positive animals on this farm cannot be excluded. The identity of the WM‐14G sequence with previously detected cattle‐derived G7 sequences from the Warmian–Masurian region supports the reliability and epidemiological plausibility of this finding. Comparative analysis of the *env* fragment from Farm 3 showed that the sequence obtained from *Culicoides* sp. shared 99.76% nucleotide identity with the cattle‐derived sequences Go0226 and Go0227 and 99.52% identity with Go0225. All sequences clustered within genotype G4, indicating the circulation of a highly similar BLV variant within this herd. BLV sequences obtained from *Culicoides* spp. were identical to those derived from cattle from Farm 4, showing 100% nucleotide identity, and all sequences were classified within genotype G4.

### 3.3. BLV DNA Detection Rates and Distribution Across *Culicoides* spp. and Gonotrophic Status

The detection rates calculated using the MDR and MLE are summarized in Table 6. The overall MLE for BLV DNA‐positive pools was estimated at 9.01 per 1000 insects (95% CI: 5.47–13.82). Farm 1 showed the highest BLV DNA detection rates, whereas no pools positive for BLV DNA were identified at Farm 2.

**Table 6:** Minimum detection rate (MDR) and maximum likelihood estimate (MLE) of BLV DNA in *Culicoides* biting midges collected on four cattle farms.

Farm	Number of pools	Number of insects tested	*Env* positive pools	MDR (per 1000 insects)	MLE (per 1000 insects)	95% CI (MLE)
Farm 1	71	1301	15	11.53	12.80	7.38–20.36
Farm 2	30	450	0	0.00	0.00	0.00–4.26
Farm 3	6	83	1	12.05	12.43	0.71–53.62
Farm 4	19	306	2	6.54	6.82	1.14–20.92
**Total**	**126**	**2140**	**18**	**8.41**	**9.01**	**5.47–13.82**

*Note:* Values are expressed per 1000 tested insects. MDR was calculated as the number of positive pools divided by the total number of tested insects. The MLE and 95% CI were estimated to account for variable pool sizes. Bold indicates the final summary result.

The distribution of BLV‐positive pools across *Culicoides* species is presented in Table 7. BLV DNA was detected in five species, with the highest number of positive pools observed in *C. punctatus* (*n* = 9), followed by *C. achrayi* (*n* = 4) and *C. obsoletus/C. scoticus* (*n* = 3). The highest MDR value was recorded for *C. fascipennis* (33.33 per 1000 insects), although it was based on a limited number of pools. No BLV‐positive pools were detected in *C. pulicaris*, *C. circumscriptus*, *C. grisescens*, or *C. newsteadi*.

**Table 7:** Detection of BLV DNA in *Culicoides* species collected on four cattle farms in Poland.

Species	Number of pools	Number of insects tested	Positive pools	MDR (per 1000 insects)
*C. punctatus*	47	876	9	10.27
*C. obsoletus/C. scoticus*	41	758	3	3.96
*C. achrayi*	15	276	4	14.49
*C. pallidicornis*	11	132	1	7.58
*C. fascipennis*	3	30	1	33.33
*C. pulicaris*	3	21	0	0.00
*C. circumscriptus*	3	28	0	0.00
*C. grisescens*	1	6	0	0.00
*C. newsteadi*	2	13	0	0.00
**Total**	**126**	**2140**	**18**	**8.41**

*Note:* Bold indicates the overall summary result.

The detection of BLV DNA varied markedly depending on gonotrophic forms (Table 8 and Supporting Information 4: Table S3). Most positive pools were associated with blood‐fed individuals, with 15 out of 23 pools (65.2%) being positive. BLV DNA was detected significantly more frequently in blood‐fed *Culicoides* pools than in nonblood‐fed pools (15/23 vs. 3/103; Fisher’s exact test, *p* = 3.15 × 10^−11^). In contrast, BLV DNA was detected only sporadically in parous (5.1%) and nulliparous (2.4%) pools and was not detected in gravid females or males.

**Table 8:** Detection of BLV DNA in *Culicoides* pools according to gonotrophic forms.

Gonotrophic status	Number of pools	Positive pools	Proportion positive (%)
Blood‐fed females	23	15	65.2
Parous females	39	2	5.1
Gravid females	18	0	0
Nulliparous females	41	1	2.4
Males	5	0	0
Total	**126**	**18**	**14.3**

*Note:* Bold indicates the overall summary result.

**Figure 4:**
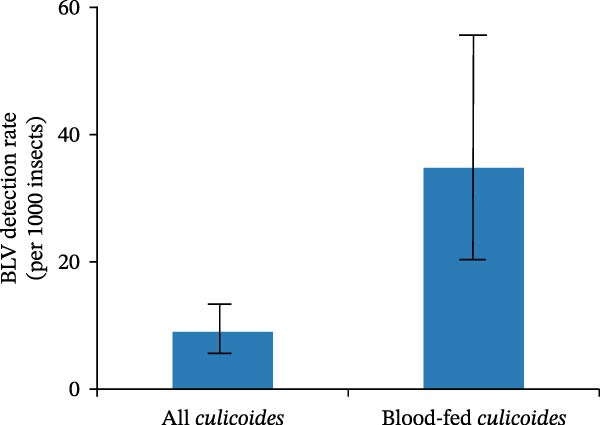
Comparison of BLV detection rates in all tested *Culicoides* pools and in blood‐fed *Culicoides* females, expressed as maximum likelihood estimates (MLE) per 1000 insects with 95% confidence intervals.

A comparison of detection rate estimates further highlighted this pattern (Figure 4). The MLE for all tested *Culicoides* pools was 9.01 per 1000 insects (95% CI: 5.47–13.82), whereas for blood‐fed females, it increased nearly fourfold to 34.78 per 1000 insects (95% CI: 21.10–57.92). The corresponding MDR values were 8.41 and 33.41 per 1000 insects, respectively. This pattern indicates that BLV detection was strongly associated with the presence of a recent blood meal.

### 3.4. *S. calcitrans* Results

All 146 *S. calcitrans* specimens were positive for the insect marker PNOC, confirming the quality of the extracted DNA. BLV DNA was detected in a single specimen (0.68%) from Farm 4, which was positive in both real‐time PCR targeting the *pol* gene and nested PCR targeting the *env* gene. The obtained sequence was included in the alignment shown in Figure 2 and was highly similar, although not identical, to the cattle‐derived sequences Go0219, Go0218, and Go0213, differing by three nucleotide substitutions within the analyzed *env* fragment and showing 99.28% nucleotide identity. Phylogenetic analysis assigned this sequence to genotype G4, consistent with the BLV strains identified in cattle from Farm 4.

## 4. Discussion

The present study provides field‐based molecular evidence that *Culicoides* biting midges and *S. calcitrans* collected on cattle farms in northeastern Poland can carry BLV proviral DNA. The most important findings registered during this study were (i) detection of BLV DNA in 18 *Culicoides* pools and one *S. calcitrans* specimen, (ii) consistent codetection of bovine DNA in all BLV‐positive *Culicoides* pools, (iii) a marked predominance of BLV‐positive results in blood‐fed pools, and (iv) complete sequence identity between insect‐derived and cattle‐derived BLV *env* sequences, all clustering within genotype G4. Together, these observations support the hypothesis that hematophagous insects may contribute to the mechanical, rather than biological, transfer of BLV between cattle, as previously suggested [1–10, 18–25].

BLV is a cell‐associated deltaretrovirus transmitted primarily through the transfer of infected lymphocytes rather than by abundant cell‐free virions [1–4]. This explains why even small quantities of blood may be epidemiologically relevant and why both iatrogenic procedures and blood‐feeding arthropods have long been considered potential contributors to transmission [1, 3, 20–28]. Experimental studies, as well as observations under natural conditions, have shown that BLV can be mechanically transferred by horse flies, horn flies, and stable flies. This has also been supported by several intervention studies suggesting that limiting exposure to biting flies may be associated with reduced on‐farm BLV transmission [20–26]. In this context, the strongest evidence supporting the proposed role of these insects in BLV mechanical transfer is the close association between BLV detection and the evidence of a bovine blood meal.

All BLV‐positive *Culicoides* pools were positive for bovine DNA, and BLV DNA was detected significantly more frequently in blood‐fed than in nonblood‐fed pools. The nearly fourfold increase of MLE among blood‐fed *Culicoides* strongly suggests that proviral DNA was acquired during feeding on infected cattle rather than through unrelated environmental contamination. This pattern fully reflects a mechanism of mechanical transfer and is in line with the broader principle that pathogens acquired with blood meals are most readily detected in recently fed individuals [20–26].

At the same time, these findings should not be interpreted as proof of the biological vector competence of *Culicoides* for BLV. In arbovirus research, detection of viral nucleic acids in field‐collected midges is widely recognized as an important epidemiological signal but is not sufficient evidence of viral replication, dissemination to the salivary glands, or effective biological transmission [5–7, 10, 29–33]. This distinction is central in studies of bluetongue virus and Schmallenberg virus, where progressively more stringent approaches, including testing of heads or head/thorax fractions, use of Ct thresholds, that is, cycle threshold values used in real‐time PCR as an indirect indicator of viral nucleic acid quantity, and laboratory infection experiments, were required to distinguish carriage of infected blood from true vector competence [29–33]. Therefore, our study demonstrates the carriage of BLV proviral DNA by field‐caught insects but does not demonstrate replication of BLV in the insect. Nevertheless, the detection of BLV DNA in *Culicoides* remains epidemiologically relevant. *Culicoides* are abundant in cattle farm environments, many species readily feed on bovines, and their veterinary importance as vectors of major ruminant viruses is well established [5–7, 29–33]. Blood meal analyses from Europe and elsewhere have shown that species such as *C. punctatus*, members of the *obsoletus* complex, and related taxa frequently feed on large mammals, including cattle [34–39]. From this perspective, even if BLV is only mechanically carried, the field codetection of bovine DNA and BLV proviral DNA in these insects is highly informative for herd‐level transmission ecology [34–39]. The species distribution observed in the present study is also noteworthy. Although all farms were located within the same wetland‐rich region, differences in *Culicoides* species composition among sites may partly reflect variation in sampling dates and seasonal dynamics of individual species. Local microhabitat conditions around cattle buildings and pastures, as well as meteorological factors preceding collection, such as temperature, precipitation, and wind speed, may also have influenced adult activity and survival. In addition, farm‐specific characteristics, including livestock density, manure handling, land use, vegetation structure, and the availability of larval habitats, could have contributed to the observed variation.

BLV DNA was detected in five *Culicoides* species, with the highest number of positive pools in *C. punctatus*, followed by *C. achrayi* and *C. obsoletus/C. scoticus*. These findings should not be interpreted as proof that one species is intrinsically a more efficient vector than another as the number of positive pools may be influenced by both the relative abundance of a given species and its opportunity for contact with BLV‐positive bovine blood. However, our data clearly show that contact with BLV‐positive bovine blood was not restricted to a single taxon. Similar reasoning has been applied in field studies of Schmallenberg virus and bluetongue virus, where several *Culicoides* species were tested positive under natural conditions, reflecting host contact patterns and ecological opportunity rather than strict vector specialization [29–33]. Another important aspect is the detection of bovine DNA in some pools that were not visibly blood‐fed. Previous blood meal studies have shown that host DNA may remain amplifiable after partial digestion of a blood meal, even when visible traces of blood have disappeared [34–39]. The mitochondrial *cyt b* marker is particularly useful in this setting because of its high copy number and greater sensitivity in partially digested samples, whereas PNOC may be less sensitive at advanced stages of digestion [35]. Thus, detection of bovine DNA in some parous, gravid, or apparently nonengorged pools is compatible with the known biology of blood meal digestion and should not automatically be interpreted as an artifact [34–39]. BLV DNA was also detected in one pool classified as nulliparous. Because nulliparous females are generally considered not to have completed a gonotrophic cycle, this result should be interpreted cautiously. The pool was also positive for bovine DNA, suggesting contact with bovine blood‐derived material. Possible explanations include the presence of a first or partially digested blood meal that was not macroscopically visible, possible misclassification of individual specimens within the pool, or low‐level contamination with bovine blood‐derived material during feeding or sample processing. Therefore, this single finding does not indicate BLV infection of nulliparous females but rather highlights the need for cautious interpretation of pooled field‐collected material.

The sequence data provide one of the strongest supports for a real epidemiological link between infected cattle and insects. Among the *pol*‐positive pools, 18 were positive in nested *env* PCR, and 15 yielded high‐quality nucleotide sequences. All insect‐derived sequences were identical to those obtained from infected cattle and all clustered within genotype G4. These findings support the local acquisition of BLV from infected bovine hosts on the studied farms [3, 8–10, 40]. Genotype G4 is one of the predominant BLV genotypes circulating in Poland and, more broadly, in Central and Eastern Europe, often together with G7 and, less frequently, G8 [8, 9, 40]. Importantly, the Turkish study that reported BLV in a *Culicoides schultzei* pool also found concordance between sequences found in insects and cattle, although in that setting, the virus belonged to genotype G1 [10]. In this respect, the present study should be regarded as the first report from Poland and to our knowledge, one in Europe linking BLV‐positive *Culicoides* with bovine host DNA and identical cattle‐derived viral sequences.

The single BLV‐positive *S. calcitrans* result is also biologically plausible and complements the *Culicoides* findings. Stable flies are classic candidates for mechanical transmission because they are persistent blood feeders, are readily interrupted by host defensive behavior, and often move rapidly between animals [21–25, 41]. Experimental work has implicated stable flies in BLV transmission, and more recent studies have shown that stable fly blood meals may reflect the BLV status of cattle herds [21–25, 41]. Although only one positive *S. calcitrans* specimen was detected in our material, this result is concordant with the existing literature and reinforces the broader interpretation that blood‐feeding Diptera may contribute to noniatrogenic BLV circulation under farm conditions [21–25, 41].

The farm‐level pattern of BLV infection is also consistent with this interpretation. The highest number of BLV‐positive *Culicoides* pools was found on Farm 1, whereas no positive pools were detected on Farm 2. This broadly mirrors the infection status of cattle on those farms and suggests that insect positivity was driven by the local presence of infected bovine hosts rather than by generalized contamination. In other words, BLV detection in insects corresponded to herd infection status, which is consistent with the hypothesis that insects acquired the virus while feeding on infected cattle [1–4, 8, 9, 40].

For a comprehensive assessment of the results, several limitations should be acknowledged. First, the study was based on pooled *Culicoides* samples, which does not allow the determination of the exact number of positive insects within each pool. Second, detection of BLV DNA does not prove infectivity or successful onward transmission. Third, the study did not distinguish virus present in ingested blood from virus potentially retained on the mouthparts. Finally, not all *pol*‐positive pools yielded interpretable *env* sequences. Despite the high overall agreement between the assays, with 18 of 20 *pol* real‐time PCR‐positive pools also positive in the *env* nested PCR, two samples were not confirmed by nested PCR. This discrepancy may be explained by methodological differences between the assays, including visual assessment of nested PCR products on agarose gels, where very weak bands may be difficult to detect. In addition, the *env* region is more variable than the *pol* gene, which may affect primer binding and amplification efficiency, as previously shown for BLV molecular assays [42].

Full‐genome amplification or sequencing of BLV from insect‐derived samples was not attempted in the present study because the main objective was to detect BLV DNA in hematophagous insects, compare partial viral sequences with cattle‐derived sequences, and determine the genotype of the detected viruses. Therefore, we used *pol* real‐time PCR and nested PCR targeting the 400‐bp *env* fragment, as these assays are recommended in the WOAH Manual and are commonly used for BLV detection and molecular characterization. Moreover, insect‐derived DNA extracts contained limited and heterogeneous material, consisting predominantly of insect DNA and host blood‐derived DNA, whereas BLV DNA copy numbers were often low and variable. Therefore, reliable amplification of the complete BLV genome from these samples would be technically challenging. This targeted approach was sufficient to confirm BLV DNA in insect‐derived samples and assign the obtained sequences to BLV genotypes. Nevertheless, full‐genome sequencing of BLV from insect‐derived samples would provide additional scientific value for molecular epidemiology, including more detailed tracking of viral circulation within the farm ecosystem, and should be considered in future studies. These limitations are typical of field‐based insect surveillance studies and do not alter the main conclusion that BLV‐positive bovine blood and BLV DNA co‐occurred in naturally collected hematophagous insects [29–33]. Despite these limitations, the epidemiological implications are highly important. Current BLV control programs focus appropriately on testing, segregation, culling, and prevention of iatrogenic blood transfer [1–4]. Our data do not challenge those priorities, but they suggest that insect exposure should not be completely ignored, particularly on farms with high host density, high insect abundance, and infected cattle with a higher proviral load or persistent lymphocytosis [1–4, 40]. Under such conditions, interrupted feeding behavior, involving sequential feeding on multiple hosts, may facilitate the mechanical transfer of BLV‐positive blood between animals [20–26, 41]. These findings also support the inclusion of insect control in cattle sheds as part of biosecurity measures.

## 5. Conclusion

This study provides the first molecular evidence from Poland that *Culicoides* biting midges and *S. calcitrans* collected on cattle farms may carry both bovine blood and BLV DNA under natural conditions. The close association between BLV detection and the presence of bovine host DNA, together with the predominance of positive results in blood‐fed pools and the complete sequence identity between insect‐ and cattle‐derived BLV strains, supports the hypothesis of mechanical transfer of BLV by hematophagous insects. Most sequenced viruses belonged to genotype G4, consistent with the dominant BLV genotype circulating in Poland, while one *Culicoides*‐derived sequence was assigned to genotype G7. These findings highlight the need for further studies on the epidemiological significance of biting insects in BLV transmission and their possible contribution to virus circulation within infected herds.

## Author Contributions

Aneta Pluta conceived the study, supervised the work, participated in data collection, laboratory analyses, data interpretation, and manuscript preparation and revision. Joanna Kowalik contributed to laboratory analyses. Wojciech Rożek and Małgorzata Kwaśnik contributed to sample collection and manuscript revision. Anna Ziętek‐Barszcz contributed to map preparation. Jacek Kuźmak contributed to manuscript revision.

## Funding

This work was supported by a subsidy from the Ministry of Science and Higher Education under Grant S/481 for research conducted in 2023 at the National Veterinary Research Institute, Puławy, Poland. The article processing charge (APC) was funded by the Institutional Basal Research Fund.

## Disclosure

All authors contributed to the article and approved the submitted version.

## Ethics Statement

Blood samples from Polish cattle, naturally infected with BLV, were selected from collections at local diagnostic laboratories as part of the EBL monitoring program in 2023 and sent to the National Veterinary Research Institute (NVRI) in Pulawy for confirmation study. The approval for collection of these samples from ethics committee was not required according to Polish regulation (“Act on the Protection of Animals Used for Scientific or Educational Purposes,” Journal of Laws of 2015). Ethical approval was not required because insects were collected.

## Conflicts of Interest

The authors declare no conflicts of interest.

## Supporting Information

Additional supporting information can be found online in the Supporting Information section.

## Supporting information


**Supporting Information 1** Table S4: DNA concentration and purity ratios (A260/A280) of *Culicoides* spp. and *Stomoxys calcitrans* pools collected from different farms.

**Supporting Information 2** Table S1: Primers used for PCR amplification of insect mitochondrial and ribosomal DNA fragments and detection of bovine and viral DNA.

**Supporting Information 3** Table S2: Characteristics of BLV sequences used in this study.

**Supporting Information 4** Table S3: Results of qPCR detection of BLV DNA and insect and bovine markers in *Culicoides* pools collected from cattle farms in Poland.

## Data Availability

The nucleotide sequences generated in this study have been deposited in the GenBank database under Accession Numbers PZ226357–PZ226361, PZ226363–PZ226366, PZ226368–PZ226369, PZ226371–PZ226375, and PZ226336–PZ226346. Additional data supporting the findings of this study are provided in the Supporting Information.
