# Supplementary material for: First Evidence of Bovine Leukemia Virus in Culicoides Biting Midges and the Stable Fly Stomoxys calcitrans Collected on Cattle Farms in Poland
Source: Transbound Emerg Dis. 2026 Jun 25;2026:7012598. doi: 10.1155/tbed/7012598 (PMC13295148; doi:10.1155/tbed/7012598)
Supplement: Supplementary file 2 — Supporting Information 2 Table S1: Primers used for PCR amplification of insect mitochondrial and ribosomal DNA fragments and detection of bovine and viral DNA. [file TBED-2026-7012598-s002.docx]

**Supplementary Table 1.** Primers used for PCR amplification of insect mitochondrial and ribosomal DNA fragments and detection of bovine and viral DNA.

| Target gene | Primer name | Sequence (5′–3′) | Expected amplicon size (bp) | Target organism | Reference |
| --- | --- | --- | --- | --- | --- |
| *Insect markers* | |  |  |  |  |
| cytochrome oxidase I (COI) | C1-J-1718 (Ron) | GGAGGATTTGGAAATTGATTAGTTC | 522 | *Culicoides* spp. | Simon et al., 1994 |
|  | C1-N-2191 (Nancy) | CCCGGTAAAATTAAAATATAAACTTC |  |  |  |
| 18S rRNA (ITS region) | NF1 (modified) | GGTGGTGCATGGCCGTTCTTAGTT | 457 | *Culicoides* spp. | Modified from Jin et al., 2022 |
|  | 18Sr2b (modified) | TACAAAGGGCAGGGACGTAAT |  |  |  |
| mitochondrial gene (COI/16S region) | N1-J-12585 | GGTCCCTTACGAATTTGAATATATCCT | 339 | *Stomoxys calcitrans* | Simon et al., 1994 |
|  | LR-N-12866 | ACATGATCTGAGTTCAAACCGG |  |  |  |
| *Bovine host markers* | |  |  |  |  |
| cytochrome b (cyt b) | cytBovine_for | GCCCCATCAAACATTTCATCATG | 362 | *Bos taurus* | This study |
|  | cytBovine_rev | GCTCCTCAGAATGATATTTGTCC |  |  |  |
| prepronociceptin (PNOC) | PNOC_for | GCATCCTTGAGTGTGAAGAGAA | 340 | *Bos taurus* | This study |
|  | PNOC_rev | TGCCTCATAAACTCACTGAACC |  |  |  |
| *BLV detection (qPCR)* | |  |  |  |  |
| BLV *pol* gene | MRBLVL Fw | CCTCAATTCCCTTTAAACTA | 120 | BLV | Rola-Łuszczak et al., 2013 |
|  | MRBLVR Rv | GTACCGGGAAGACTGGATTA |  |  |  |
|  | MRBLV Probe | 6FAM-GAACGCCTCCAGGCCCTTCA-BHQ1 |  |  |  |
| *BLV env nested PCR* | |  |  |  |  |
| BLV *env* gene | env5032 | TCTGTGCCAAGTCTCCCAGATA | 444 | BLV | Beier et al., 2001 |
|  | env5608 | AACAACAACCTCTGGGAAGGGT |  |  |  |
|  | env5099 | CCCACAAGGGCGGCGCCGGTTT |  |  |  |
|  | env5521 | GCGAGGCCGGGTCCAGAGCTGG |  |  |  |
